# The autophagic protein FYCO1 controls TNFRSF10/TRAIL receptor induced apoptosis and is inactivated by CASP8 (caspase 8)

**DOI:** 10.1080/15548627.2023.2229656

**Published:** 2023-07-07

**Authors:** Valeria Coppola, Ilaria Marino, Uwe Warnken, Mario Falchi, Luca Pasquini, Mauro Biffoni, Ruggero De Maria, Tobias Longin Haas

**Affiliations:** a Department of Oncology and Molecular Medicine, Istituto Superiore di Sanità, Rome, RM, Italy; b Department of Translational Medicine and Surgery, Università Cattolica Del Sacro Cuore, Rome, RM, Italy; c Functional Proteomic Analysis, German Cancer Research Center (DKFZ), Heidelberg, BW, Germany; d Clinical Cooperation Unit Neurooncology, German Cancer Research Center (DKFZ), Heidelberg, BW, Germany; e National AIDS Center (CNAIDS), Rome, RM, Italy; f Servizio Tecnico Scientifico Grandi Strumentazioni E Core Facilities – FAST, Rome, RM, Italy; g Fondazione Policlinico Universitario A. Gemelli IRCCS, Rome, RM, Italy; h Section of Immunotherapy, IIGM-Italian Institute for Genomic Medicine, Candiolo, TO, Italy

**Keywords:** apoptosis, autophagy, caspase 8, FYCO1, lysosomal degradation, TRAIL

## Abstract

Apoptosis is a tightly controlled cell death program executed by proteases, the so-called caspases. It plays an important role in tissue homeostasis and is often dysregulated in cancer. Here, we identified FYCO1, a protein that promotes microtubule plus end-directed transport of autophagic and endosomal vesicles as a molecular interaction partner of activated CASP8 (caspase 8). The absence of FYCO1 sensitized cells to basal and TNFSF10/TRAIL-induced apoptosis by receptor accumulation and stabilization of the Death Inducing Signaling Complex (DISC). Loss of FYCO1 resulted in impaired transport of TNFRSF10B/TRAIL-R2/DR5 (TNF receptor superfamily member 10b) to the lysosomes in TNFSF10/TRAIL-stimulated cells. More in detail, we show that FYCO1 interacted via its C-terminal GOLD domain with the CCZ1-MON1A complex, which is necessary for RAB7A activation and for the fusion of autophagosomal/endosomal vesicles with lysosomes. We demonstrated that FYCO1 is a novel and specific CASP8 substrate. The cleavage at aspartate 1306 resulted in the release of the C-terminal GOLD domain, inactivating FYCO1 function, and allowing for the progression of apoptosis. Furthermore, the lack of FYCO1 resulted in a stronger and prolonged formation of the TNFRSF1A/TNF-R1 signaling complex. Thus, FYCO1 limits the ligand-induced and steady-state signaling of TNFR-superfamily members, providing a control mechanism that fine-tunes both apoptotic and inflammatory answers.

**Abbreviations**: AP: affinity purification; CHX: cycloheximide; co-IP: co-immunoprecipitation; CRISPR: clustered regularly interspaced short palindromic repeats; DISC: death-inducing signaling complex; DR: death receptors; doxy: doxycycline; GEF: guanine nucleotide exchange factor; ind: inducible; KD: knockdown; KO: knockout; MS: mass spectrometry; shRNA: short hairpin RNA; siRNA: small interfering RNA; TIP: two-step co-immunoprecipitation; WB: western blot.

## Introduction

Multicellular organisms have evolved complex mechanisms to preserve homeostatic cell numbers despite of incoming proliferation or cell death signals. Apoptosis is a “suicide” process that enables the silent and controlled elimination of superfluous cells during development. It guarantees adequate homeostatic turnover in proliferating tissues and the removal of malfunctioning or damaged cells in the adult organism [1]. Defects in apoptosis cause cancer, autoimmune disease, or sustained viral infection, while excessive cell death is found in neurodegenerative disorders, heart disease, and immunodeficiencies [2]. Stimulation of death receptors (DRs) [3] belonging to the Tumor Necrosis Factor (TNF) receptor superfamily, such as FAS/CD95 (Fas cell surface death receptor) or receptors of TNFSF10/TRAIL (TNF-related apoptosis-inducing ligand), result in the formation of a Death-Inducing Signaling Complex (DISC). This leads to the activation of the initiator CASP8 (caspase 8), which can propagate the apoptosis cascade by direct cleavage of downstream effector caspases [4]. ProCASP8 is expressed as a zymogen and its activation requires binding to the adapter protein FADD (Fas-associated via death domain), which is recruited to trimerized DRs via its Death Domain. Subsequent oligomerization of proCASP8 via Death Effector Domains leads to autoproteolytic sequential cleavages that release the fully mature and active CASP8 [5].

FYCO1 (FYVE and coiled-coil domain autophagy adaptor 1) is a protein described to be involved in macroautophagy/autophagy. Autophagy is a “self-eating” catabolic process by which double-membrane vesicles called “autophagosomes” engulf proteins and damaged organelles, these are delivered to lysosomes via cytoskeletal routes where their contents are broken down to metabolic precursors and recycled back to the cytosol [6]. Thereby, the autophagic process offers a vital cytoprotective mechanism that is especially engaged during nutrient deprivation but also occurs under nutrient-rich conditions, ensuring physiological renewal of cellular components, as well as scavenging of harmful material such as aggregation-prone misfolded proteins, intracellular pathogens, and immune-related or inflammatory molecules [7,8]. FYCO1 was reported to promote microtubule plus end – directed transport of autophagic vesicles by acting as an adaptor protein between the autophagic marker MAP1LC3B/LC3B (microtubule associated protein 1 light chain 3 beta) and RAB7A (RAB7A, member RAS oncogene family) [9,10], that binds via FYCO1 to the motor proteins kinesins [11]. The same machinery is implicated in endosomal vesicle transport [11–13].

Here, we have identified FYCO1 as a molecular interaction partner of CASP8 in cells stimulated with TNFSF10/TRAIL. Using RNA interference and CRISPR-Cas9-based loss of function strategies, we found that the absence of FYCO1 sensitized cells to DR-induced apoptosis via stabilization of the DISC, caused by defective lysosomal maturation. In turn, cleavage of FYCO1 by CASP8 inactivated its function and allowed the progression of the apoptotic cascade upon a critical duration or intensity threshold of the death signal. Finally, we show that the lack of FYCO1 also resulted in a more efficient and prolonged formation of the TNFRSF1A/TNF-R (TNF receptor superfamily member 1A) signaling complex, suggesting widespread implications also for TNF-induced inflammatory answers.

## Results

### FYCO1 interacts with and is cleaved by activated CASP8

**Figure 1.**
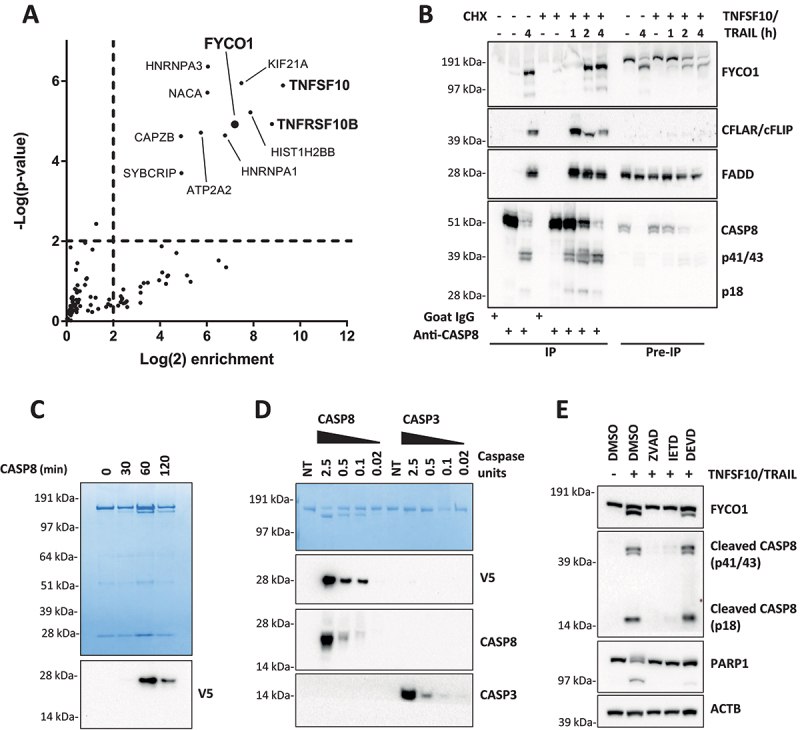
FYCO1 interacts with and is cleaved by activated CASP8. (A) Scatterplot representing proteins enriched upon CASP8 affinity isolation by mass spectrometry analysis. HeLa treated with TNFSF10/TRAIL were compared with untreated cells. The cut off for significant enrichment was set at 4-fold label-free quantitation (LFQ) values and a p-value of < 0.01 (-log (p-value) > 2). (B) WB of lysates and anti-CASP8 co-IP of HeLa cells treated with TNFSF10/TRAIL (500 ng/ml) and CHX (1 µg/ml) for the indicated timeframes. The WB was performed for the proteins indicated. (C) In vitro CASP8 cleavage assay. Coomassie Brilliant Blue staining of recombinant FYCO1-V5 followed by incubation with 1 U recombinant CASP8. Below, the WB for anti-V5. (D) In vitro CASP8 and CASP3 cleavage assays. Coomassie blue staining of recombinant FYCO1-V5 followed by incubation for 1 h with a titration of recombinant caspase units. Below, the WB for the antibodies indicated. (E) WB of HeLa lysates treated with TNFSF10/TRAIL (1 µg/ml, 2 h) in combination with different caspase inhibitors (20 µM).

To search for binding partners of activated CASP8, we induced apoptosis with TNFSF10/TRAIL and performed two-step co-immunoprecipitation (TIP) using a polyclonal anti-CASP8 antibody [14]. TIP is a novel biochemical method that, by means of a biotin-based tandem purification, greatly facilitates the identification of novel molecular interactions [15,16]. The proteins enriched in CASP8 precipitates of unstimulated and TNFSF10/TRAIL-stimulated HeLa cells were analyzed by mass spectrometry (MS). Compared to the isotype control, RIPK1 (receptor-interacting serine/threonine kinase 1), CASP8, CASP10 (caspase 10), and CFLAR/cFLIP (CASP8 and FADD-like apoptosis regulator) were significantly enriched upon CASP8 TIP independent of the stimulation (see **Table S1**). In the CASP8 interactome of the TNFSF10/TRAIL-treated cells, peptides of 11 additional proteins were significantly enriched (Figure 1A). These “hits” were matched to the CRAPome repository, the biggest database aimed to collect and publish nonspecific protein identifications in mass spectrometry analyses. While most of the identified proteins are commonly found in affinity purification (AP) experiments, only TNFSF10/TRAIL, its receptor TNFRSF10B/TRAILR2/DR5 (TNF receptor superfamily member 10b), and FYCO1 were identified in less than 1% of the tandem AP experiments collected in the CRAPome database, making a random enrichment unlikely (**Table S2**). Immunoblot experiments confirmed that, in presence of TNFSF10/TRAIL, the CASP8 co-immunoprecipitation (co-IP) resulted in a pull-down of FYCO1 along with established interaction partners FADD and CFLAR/cFLIP in a cycloheximide (CHX)-independent manner (Figure 1B). Interestingly, the western blot (WB) for FYCO1 showed two signals of different molecular weights in the lysates of TNFSF10/TRAIL-stimulated cells, while the precipitated form of FYCO1 was exclusively the fragment of lower molecular weight (Figure 1B). These results suggested that FYCO1 was cleaved upon stimulation with TNFSF10/TRAIL and that only the cleaved fragment was enriched in the anti-CASP8 co-IP. The presence of FYCO1 in anti-CASP8 co-IPs was confirmed upon stimulation of cells with different death ligands known to activate CASP8 (FASLG, TNF, TNFSF10/TRAIL) (**Figure S1A**). In our HeLa cellular model, TNFSF10/TRAIL provided the strongest CASP8 activation; thus, we continued to investigate the function of FYCO1 using this stimulus. By performing a reverse co-IP with a polyclonal anti-FYCO1 antibody, we validated the co-immunoprecipitation of the activated CASP8 (p18) in the TNFSF10/TRAIL-stimulated sample (**Figure S1B**).

Since the cleavage of FYCO1 correlated with caspase activation, we performed an *in vitro* cleavage assay with purified FYCO1 carrying a C-terminal V5-tag. As observed by western blot analyses in apoptotic cell lysates, the sodium dodecyl sulfate – polyacrylamide gel electrophoresis (SDS-PAGE) revealed the appearance of an FYCO1 doublet after 1 h of incubation with active CASP8 (Figure 1C). Moreover, an immunoblot using anti-V5 showed that the cleavage gave rise to a C-terminal fragment of approximately 28 kDa (Figure 1C). We then compared the activity of recombinant human CASP8 and CASP3 (caspase 3). CASP8 cleaved FYCO1 efficiently even at low concentrations. In contrast, CASP3 showed only minimal cleavage activity on recombinant FYCO1 even when used at significantly higher concentrations (Figure 1D). To avoid any experimental bias, we tested the specific activity of the recombinant enzymes for their respective substrates and found that they were comparable (**Figure S1C**). Thus, we assumed that FYCO1 was cleaved by CASP8 upon its activation with high specificity *in vitro*. The assumption that in cells the cleavage is mediated by CASP8 was also supported by the observation that pre-incubation of the cells with pan-caspase (Z-VAD-FMK) and CASP8 inhibitors (Z-IETD-FMK), but not with CASP3 inhibitor (Z-DEVD-FMK), prevented FYCO1 cleavage upon TNFSF10/TRAIL treatment (Figure 1E). Importantly, while the active CASP8 was generated in presence of Z-DEVD-FMK, the cleavage of the executioner caspase substrate PARP was largely inhibited under these conditions. In summary, these experiments demonstrated that FYCO1 is a novel substrate of CASP8.

### FYCO1 knockdown sensitizes cells to basal and DR-induced apoptosis.

**Figure 2.**
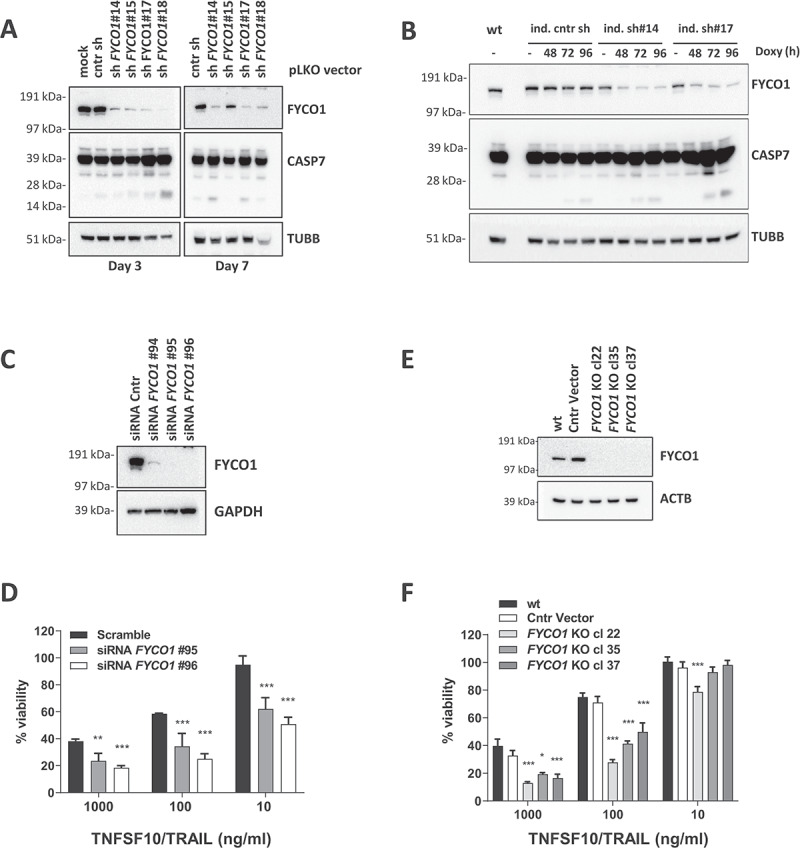
FYCO1 KD sensitizes cells to basal and DR-induced apoptosis. (A) HeLa cells were transduced with 4 different shRNAs for FYCO1 (#14; #15; #17 and #18) and control. Lysates were prepared after 3 or 7 days and WB analyses for the indicated proteins were performed. (B) FYCO1 expression in HeLa cells was knocked down with 2 different inducible shRNAs (ind. sh#14 and #17) by the addition of doxycycline (250 ng/ml) for the timeframes shown. Afterwards, WB analyses for the proteins indicated were performed. (C) WB to validate FYCO1 KD by 3 different siRNA oligos (40 nM, 72 h). (D) Cell viability assay (Cell titer Glo) of HeLa cells knocked down for FYCO1 by siRNA (40 nM, standard error of the average viability (*n* = 3). Statistical analysis was performed by two-way ANOVA with Dunnett test vs scramble control. (E) WB to validate FYCO1 knockout by CRISPR-Cas9 mediated gene editing. (F) Cell viability assay (Cell titer Glo) of control and FYCO1 knockout HeLa cells as in (E), subjected to titration of TNFSF10/TRAIL treatment for 24 h. Reported is the mean ± standard error of the average viability (*n* = 3). Statistical analysis was performed by two-way ANOVA with Dunnett test vs WT cells.

We investigated the biological effect of FYCO1 knockdown (KD) in HeLa cells with four different constitutive short hairpin RNA (shRNA) constructs. *FYCO1* shRNAs induced activation of the caspase cascade, shown by cleavage of CASP7 (caspase 7), with different potency and timing of the construct. The effect of shRNA #18 became apparent at 3 days whereas shRNAs #14 and #17 needed 7 days. The control and a nonfunctional *FYCO1* shRNA were not inducing significant caspase cleavage (Figure 2A). The apoptotic phenotype, including morphological changes of the cells and blebbing of the membranes, was fully reverted by the pan-caspase inhibitor Z-VAD-FMK (**Figure S2A**). To reduce the impact of potential apoptotic stimuli deriving from cell stress due to shRNA transduction, cells carrying two independent doxycycline-inducible shRNA constructs were engineered. In this setting, an efficient FYCO1 KD after 72 to 96 h also resulted in caspase activation (Figure 2B).

Similar results were obtained by small interfering RNA (siRNA)-mediated FYCO1 silencing. Three different siRNAs were tested and the two most efficient were selected (Figure 2C). As before, FYCO1 interference resulted in cell loss that was rescued by co-incubation with the pan-caspase inhibitor Z-VAD-FMK and the CASP8 inhibitor Z-IETD-FMK (**Figure S2B**). However, because we found that at least 50% of the cells were alive and adherent 72 h post-transfection, we tested them for sensitivity to extrinsic apoptosis stimuli known to induce CASP8 activation: TNFSF10/TRAIL, FASLG, TNF, and polyinosinic:polycytidylic acid (Poly IC). Independently of the strength of the stimulus, which was strongest with TNFSF10/TRAIL, FYCO1 KD resulted in enhanced caspase cleavage (**Figure S2C**). Cell viability assays confirmed a significant sensitization to TNFSF10/TRAIL with both siRNAs (Figure 2 2D). Interestingly, siRNA and shRNA-mediated knockdown of FYCO1 also sensitized resistant T47D breast carcinoma cells [17,18] to TNFSF10/TRAIL-induced caspase activation and cell loss (**Figure S2D-E**), suggesting that FYCO1 neutralization can also sensitize primarily resistant cells to the death ligand. Thus, FYCO1 might act as an apoptosis inhibitor.

To further corroborate these results, and to obtain a stable working system for the investigation of the biochemical mechanisms, we proceeded with a CRISPR-Cas9-mediated gene editing of FYCO1 taking advantage of the pX330-U6-Chimeric_BB-CBh-hSpCas9 vector [19]. Although transfection with FYCO1 gene targeting single guide RNAs (sgRNAs) resulted in pronounced cell death, we obtained stable and proliferating clones of HeLa cells with FYCO1 knockout (KO) (Figure 2E). In line with the previous results, these cells were more sensitive to TNFSF10/TRAIL-induced apoptosis (Figure 2F and **Figure S2F**). To further rule out the possibility of cell line-specific phenomena, these results were confirmed in HCT116 colon carcinoma cells subjected to shRNA interference (**Figure S3A**) and CRISPR-Cas9-mediated gene knockout of FYCO1 (**Figures S3B-D**). The gene editing was controlled by Sanger sequencing of the genetic loci targeted by the sgRNAs and functional knockouts in 50–100% of the alleles were validated (**Figure S3E**). Interestingly in only one of the clones (HeLa cl.37) all 30 sequencing reactions identified frameshift mutations and thus a complete functional knockout.

These results suggest that in most clones a low background expression of FYCO1 is needed for cell survival or proliferation and that western blot analyses are not sensitive enough to detect the remaining protein. Although the genomic KO was not 100%, we describe the CRISPR-edited clones as KO throughout the manuscript as we assume that most of the biological functionality of FYCO1 was lost in these cells.

### The absence of FYCO1 leads to the stabilization of the DISC caused by impaired lysosomal degradation

**Figure 3.**
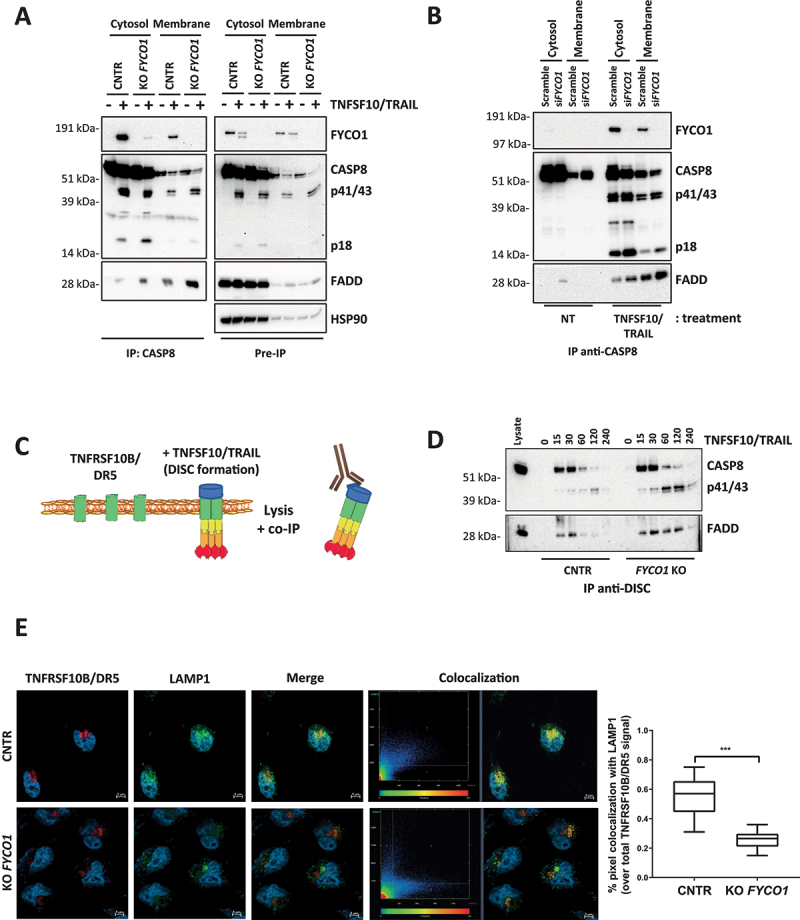
The absence of FYCO1 leads to the stabilization the DISC caused by impaired lysosomal degradation. (A) Cytosolic and membrane fractions of HeLa cells knocked out for FYCO1 by CRISPR-Cas9 and treated with TNFSF10/TRAIL (2 h, 500 ng/ml) were prepared. Anti-CASP8 co-IP was performed. Shown is the WB analysis for the proteins indicated. (B) Cytosolic and membrane fractions of HeLa cells transfected with scramble or siRNA for FYCO1 (#95, 40 nM, 72 h) treated or not with TNFSF10/TRAIL (500 ng/ml, 2 h) were prepared and anti-CASP8 co-IPs were performed. Shown are the WB tested for the proteins indicated. (C) Schematic representation of the DISC isolation. (D) WB analysis of the TNFSF10/TRAIL DISC. Cells were stimulated with 500 ng/ml FLAG-tagged TNFSF10/TRAIL for the indicated timeframes. The receptor-associated proteins were then immunoprecipitated with anti-FLAG agarose and WB analysis for the proteins indicated was performed. (E) TNFRSF10B/DR5 localization assay. Control and KO FYCO1 HeLa cells were treated with bafilomycin A_1_ (1 h, 100 nM) and then with TNFSF10/TRAIL (2 h, 1 µg/ml). Representative immunofluorescence showing TNFRSF10B/DR5 (red) and LAMP1 (green) staining (63X magnification). Colocalization is highlighted in yellow and represented by a scatter plot of pixels in the two fluorescence channels. The percentage of TNFRSF10B/DR5 colocalization with LAMP1 was calculated for 10 representative fields and used for box plot graphical representation and statistical analysis by Student t-test.

To decipher how FYCO1 affected caspase activation, cytoplasmic and membrane fractions of control and *FYCO1* KO cells were prepared, and anti-CASP8 co-IPs were performed. Interestingly, in the absence of FYCO1, we observed more recruitment of the death effector protein FADD, especially in the membrane compartment (Figure 3A). We validated this result upon siRNA-mediated FYCO1 silencing (Figure 3B). Then, we focused on the signaling initiated at the plasma membrane and analyzed the composition of the TNFSF10/TRAIL DISC that is formed upon the stimulation of living cells with TNFSF10/TRAIL (Figure 3C). In *FYCO1* KO cells we observed a significant stabilization of the DISC for up to 120 min, while in control cells the association between CASP8 and FADD declined after 30 min (Figure 3D).

Since the internalization of the TNFSF10/TRAIL receptors plays an important role in their function [20] and FYCO1 was described as part of the molecular machinery implicated in autophagosomal and endosomal vesicle transport [11–13], we hypothesized that the absence of FYCO1 could influence the signaling by hampering lysosomal degradation of the TNFSF10/TRAIL receptors. Consistent with this hypothesis, immunofluorescence-based analysis of control and *FYCO1* KO cells treated with TNFSF10/TRAIL and with bafilomycin A_1_ to prevent lysosomal acidification showed that colocalization of TNFRSF10B/DR5 with the lysosomal marker LAMP1 (lysosomal associated membrane protein 1) was significantly reduced in the absence of FYCO1 (Figure 3E).

### Cleavage of FYCO1 occurs at Aspartate 1306 and blunts its function

**Figure 4.**
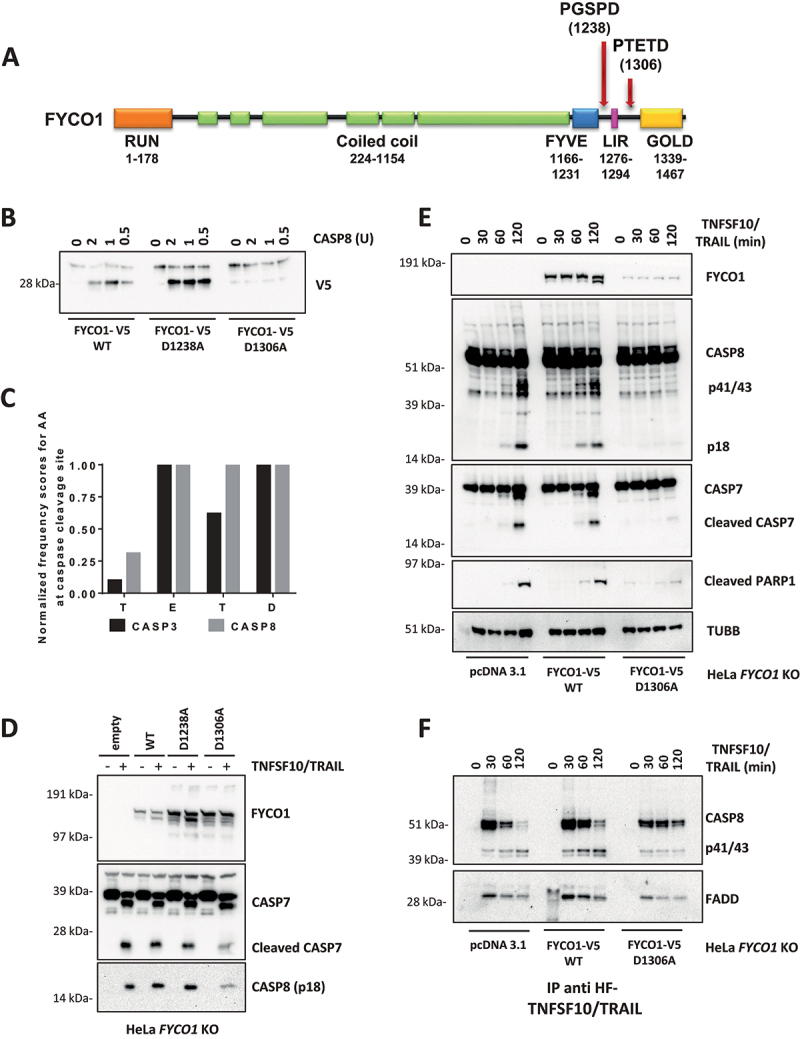
Cleavage of FYCO1 occurs at Aspartate 1306 and blunts its function. (A) Scheme of the FYCO1 protein structure. Arrows indicate two putative CASP8 cleavage sites. (B) In vitro CASP8 cleavage assay. Bead-bound FYCO1 WT-V5, FYCO1^D1238A^-V5, and FYCO1^D1306A^-V5 were incubated for 1 h with the indicated concentrations of recombinant CASP8. Then a WB detecting the V5 epitope was performed. (C) The TETD tetrapeptide is a preferential cleavage site for CASP8. The graph was obtained by plotting the normalized frequency scores of each aa at the CASP3 and CASP8 cleavage sites, as obtained by the SitePrediction web tool. (D) KO FYCO1 cells were transfected with empty pcDNA 3.1 vector or plasmids encoding FYCO1 WT, FYCO1^D1238A^, or FYCO1^D1306A^. Then cells were treated with TNFSF10/TRAIL (500 ng/ml, 2 h), lysed and a WB for the proteins indicated was performed. (E) HeLa KO FYCO1 were transfected with empty pcDNA 3.1 vector or plasmids encoding FYCO1 WT, or FYCO1^D1306A^. Then cells were treated with 500 ng/ml FLAG-tagged TNFSF10/TRAIL as indicated. Lysates were tested by WB for the indicated proteins. (F) Cells were treated as in (E) and the lysates were subjected to anti-DISC immunoprecipitation by anti-FLAG beads. Shown is a representative WB analysis for the protein indicated.

We proceeded to investigate where FYCO1 was cleaved by activated CASP8 and whether this cleavage had a biological function. Based on consensus sequences and on the molecular weight of the C-terminal fragment, we focused our attention on two potential CASP8 cleavage sites: D1238 and D1306 (Figure 4A). We performed site-directed mutagenesis of these aspartates and tested the resulting proteins in *in vitro* cleavage assays. In contrast to FYCO1 wild type (WT) and the mutant FYCO1^D1238A^, FYCO1 mutated at aspartate 1306 (FYCO1^D1306A^) was not processed by recombinant CASP8 (Figure 4B), indicating that the cleavage site was behind D1306, namely at the tetrapeptide TETD. According to the SitePrediction tool [21], the frequency of these amino acids identifies this peptide as a preferential cleavage site for CASP8, although, due to the overlap in substrate specificity, some cleavage by CASP3 cannot be ruled out (Figure 4C). Then, wild-type and mutant variants of FYCO1 were re-expressed in KO cells, and we observed that only the non-cleavable FYCO1^D1306A^ reduced the activation of caspases upon TNFSF10/TRAIL treatment (Figure 4D,E). When compared to KO cells transfected with the control vector or FYCO1 WT, we observed a reduced DISC formation in *FYCO1* KO cells reconstituted with the non-cleavable FYCO1^D1306A^ mutant (Figure 4F). Therefore, we argued that FYCO1 cleavage represents a mechanism to inactivate the protein during the induction of apoptosis.

### The C terminus of FYCO1 interacts with CCZ1

**Figure 5.**
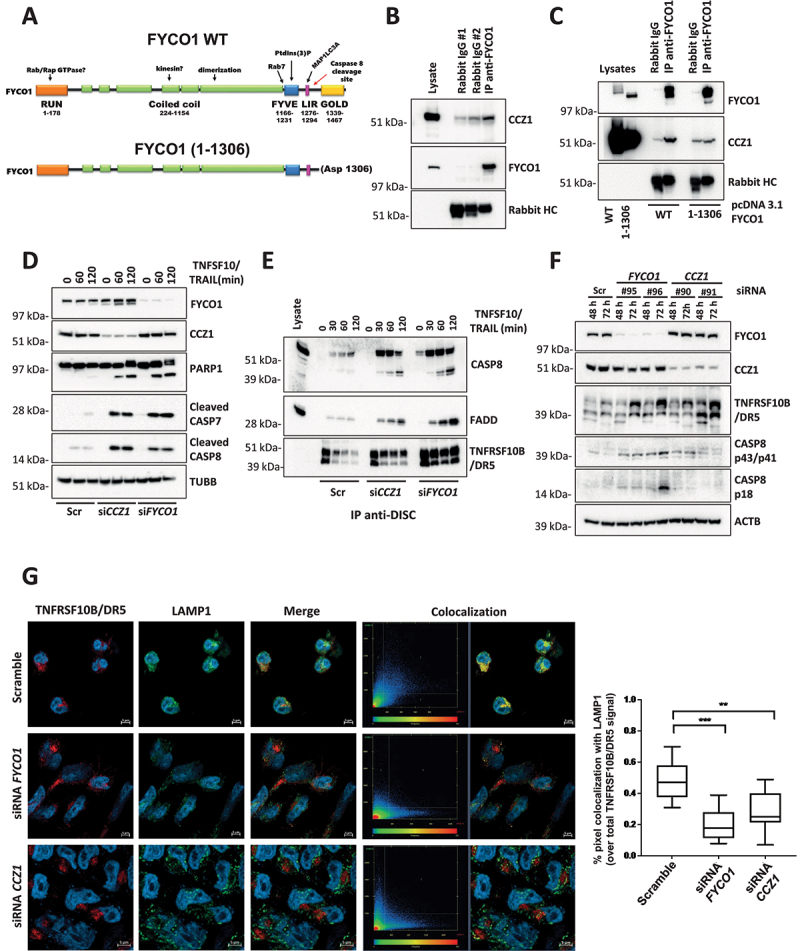
The C terminus of FYCO1 interacts with CCZ1. (A) Scheme of FYCO1 WT protein and of the truncated FYCO1 1–1306. (B) Cells were treated with 500 nM rapamycin (4 h), lysed and anti-FYCO1 or anti-rabbit IgG (isotype control) co-IPs were performed. Shown is a WB for the proteins indicated. (C) HeLa KO FYCO1 were reconstituted with either FYCO1 WT or its truncated version (1–1306), pre-treated with rapamycin as in (B), lysed and co-IP with anti-FYCO1 antibody was performed. Shown is the WB for the proteins indicated. (D) HeLa cells were transfected with the siRnas indicated (40 nM, 72 h), treated with 500 ng/ml TNFSF10/TRAIL, lysed, and WB for the proteins indicated was performed. (E) Lysates were prepared as in (D) and subjected to anti-DISC immunoprecipitation using anti-FLAG beads. The precipitated proteins were analyzed by WB with the antibodies indicated. (F) HeLa cells were transfected with two different siRNAs targeting FYCO1 and CCZ1. Cells were lysed after 48 and 72 h and WB analysis was performed for the indicated proteins. (G) TNFRSF10B/DR5 localization assay. HeLa cells were transfected with scramble, siRNA targeting FYCO1 (#96) or CCZ1 (#91) at 40 nM for 72 h. Afterward, they were pre-treated with bafilomycin A_1_ (1 h, 100 nM) and then with TNFSF10/TRAIL (2 h, 1 µg/ml). Representative immunofluorescence showing TNFRSF10B/DR5 (red) and LAMP1 (green) staining (63X magnification). Colocalization is highlighted in yellow and represented by a scatter plot of pixels in the two fluorescence channels. The percentage of TNFRSF10B/DR5 colocalization with lysosomes was calculated for 10 representative fields and used for box plot graphical representation and statistical analysis by Student t-test.

Since the non-cleavable FYCO1^D1306A^ mutant was able to protect from TNFSF10/TRAIL-induced cell death, we reasoned that the cleavage of the C-terminal region of FYCO1 may result in loss of function due to impaired interaction with other proteins. FYCO1 is a multidomain protein [22] containing an N-terminal RUN domain; a central coiled-coil region responsible for FYCO1 dimerization and interaction with RAB7A and kinesins; an FYVE domain defined as a Phosphatidylinositol 3-phosphate (PtdIns3P) binding module, necessary for FYCO1 targeting to membranes; the LC3-interacting region (LIR) motif that is important for membrane targeting through LC3B binding; and a C-terminal Golgi dynamics (GOLD) domain (Figure 5A). Cleavage at D1306 leaves the LIR motif intact, suggesting that this domain may not be the primary target of protein loss of function. Indeed, in a reconstitution experiment in which we ectopically expressed an FYCO1^F1280A,I1283A^ mutant with defective LIR unable to bind LC3 and to mediate autophagosome maturation [23] **(Figure S4A)**, we only observed a slightly higher production of active CASP8 and of the cleaved PARP product **(Figure S4B)**.

The GOLD-domain is located downstream of the CASP8 cleavage site and was reported to interact with the CCZ1 (CCZ1 homolog, vacuolar protein trafficking and biogenesis associated)-MON1A (MON1 homolog A, secretory trafficking associated) complex. This acts as a Guanine nucleotide-Exchange Factor (GEF) necessary to activate RAB7A, thereby allowing endosome/autophagosome maturation and fusion with lysosomes [24–28]. We confirmed the interaction between endogenous FYCO1 and CCZ1 in cells stimulated with the autophagy inducer rapamycin to induce FYCO1 redistribution (Figure 5B). In *FYCO1* KO cells reconstituted with a truncated FYCO1^1–^^1306^ mimicking the caspase-cleaved protein, this interaction was lost (Figure 5C). Interestingly, siRNA-mediated KD of CCZ1 phenocopied the effect of FYCO1 interference, with a strong sensitization for TNFSF10/TRAIL-induced caspase activation (Figure 5D) and the stabilization of the DISC. Noteworthy, along with an accumulation of activated CASP8 and FADD, we observed an enrichment of TNFRSF10B/DR5 in the DISC over time when either CCZ1 or FYCO1 were absent (Figure 5E). In cell lysates, an accumulation of TNFRSF10B/DR5 was detected upon loss of FYCO1 or CCZ1. This correlated with the auto-activation of CASP8 independent of any extrinsic death signal (Figure 5F). The cytofluorimetric analysis confirmed an increase of TNFRSF10B/DR5 at the cell surface of cells with FYCO1 KD, while none of the other three receptors binding TNFSF10/TRAIL (TNFRSF10A/TRAIL-R1/DR4, TNFRSF10C/TRAIL-R3/DcR1, TNFRSF10D/TRAIL-R4/DcR2) showed increased expression (**Figure S5A**). TNFRSF10B/DR5 was also increased by FYCO1 knockdown in T47D cells (**Figure S5B**). These data suggest that TNFRSF10B/DR5 may be the primary receptor for TNFSF10/TRAIL in our cell system. In line with the results obtained with *FYCO1* KO cells, both FYCO1 or CCZ1 interference were associated with a reduced colocalization of TNFRSF10B/DR5 and the lysosomal marker LAMP1, in cells treated with TNFSF10/TRAIL and bafilomycin A_1_ (Figure 5G).

To further confirm this mechanism, we silenced two more proteins important for endosome-lysosome fusion. Upon siRNA-mediated knockdown of RAB7A and VPS41 (Vacuolar Protein Sorting-Associated Protein 41 Homolog) (**Figure S5C)**, which is a core component of the HOPS complex involved in heterotypic fusions between late endosomes and lysosomes for degradation of endocytosed cargo [29], we observed a higher sensitivity to TNFSF10/TRAIL stimulation (**Figure S5D**) and an increase of TNFRSF10B/DR5 expression on the cell surface (**Figure S5E-F**). However, these effects were less pronounced as with FYCO1 knockdown.

Altogether, albeit not excluding other possible mechanisms contributing to the observed sensitization, these results indicated that in the absence of a functional endo-lysosomal machinery, degradation of the DISC was impaired, resulting in prolongation and amplification of the apoptotic signal.

### The C-terminal cleavage fragment of FYCO1 impairs FYCO1 binding to CCZ1

**Figure 6.**
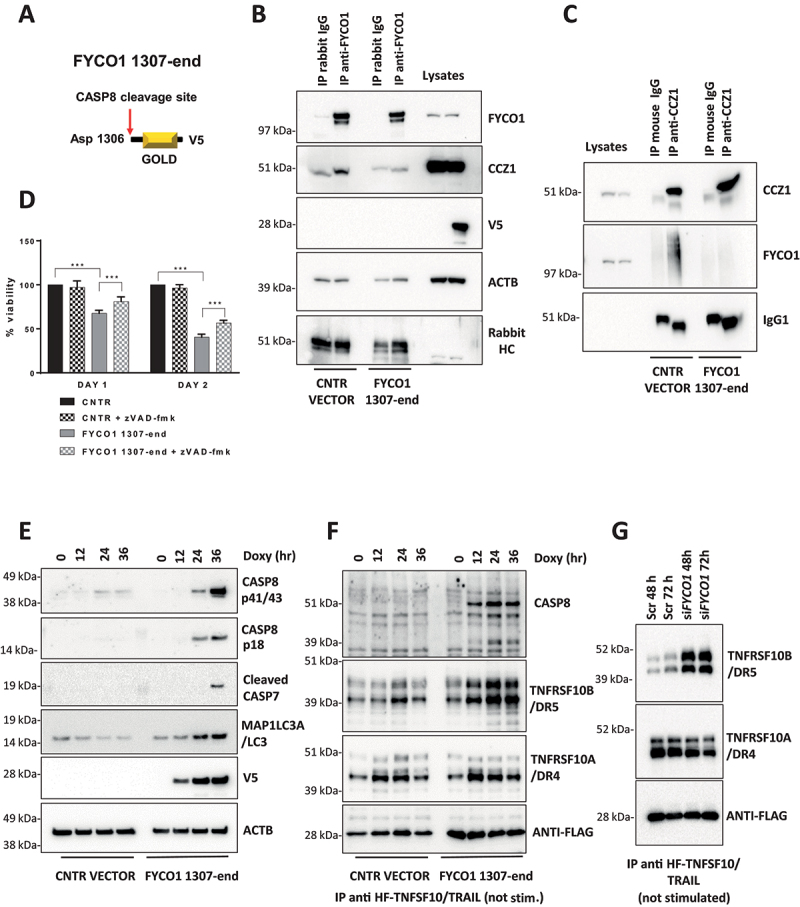
The C-terminal cleavage fragment of FYCO1 impairs FYCO1 binding to CCZ1. (A) Graphical scheme of FYCO1 C-terminal fragment (1307-end), V5-tagged and cloned into a doxycycline-inducible vector. (B) HeLa were transduced with either empty or FYCO1 1307-end encoding vector, induced by doxycycline (18 h, 250 ng/ml) and treated with the autophagy inducer rapamycin (500 nM, 4 h); then, anti-FYCO1 and rabbit IgG Co-IPs and WB for the indicated proteins was performed. (C) Samples were prepared as in (B). Anti-CCZ1 and control IgG1 Co-IPs and WB for the indicated proteins was performed. (D) HeLa transduced with either empty or FYCO1 1307-end encoding vector were induced for one or two days with doxycycline, in presence or not of Z-VAD-FMK (20 µM). Mean ± standard deviation of 4 experiments, statistical analysis by Two-way ANOVA with Tukey’s multiple comparison test. (E) Ectopic expression of FYCO1 C-terminal fragment was induced by doxycycline treatment (75 ng/ml, 12-24-36 h), then the cells were lysed and WB for the indicated proteins was performed. (F) Cells were treated as in (E). Then the samples were immunoprecipitated. The associated proteins were visualized by immunoblotting. (G) Receptor-associated proteins were isolated from cells depleted for FYCO1 expression by siRNA (#96) mediated KD for 48 and 72 h, then analyzed by immunoblotting.

We reasoned that the association of FYCO1 and CCZ1 may be responsible for the removal of stochastic complexes of TNFRSF10B/DR5 and CASP8 that result in protease auto-activation and cell death. To verify this hypothesis, we aimed to obtain a dominant-negative inhibition and ectopically expressed a V5-tagged version of the C-terminal GOLD-domain-containing FYCO1 cleavage fragment (aa 1307-end) in a doxycycline-inducible manner (Figure 6A). Under these conditions, reciprocal co-IP experiments showed a loss of FYCO1-CCZ1 interaction (Figures 6B,C). Strikingly, induction with minimal doses of doxycycline was associated with notable cell death over time, which was only partially rescued by Z-VAD-FMK treatment (Figure 6D). Additional cell death was not rescued by necrostatin (data not shown) and might be due to perturbance of physiologic lysosomal function, demonstrated by strong LC3B-II accumulation (**Figure S6A**), similarly observed in *FYCO* KO cells (**Figure S6B**). Of note, the C-terminal FYCO1 fragment was localized in the cytosol and not in membrane compartments like mitochondria, thus excluding an effect on apoptosis due to the perturbance of mitochondrial polarization (**Figure S6C**). Interestingly, the ectopic expression of the C-terminal FYCO1 fragment was associated with a strong accumulation of TNFRSF10B/DR5 and ligand-independent activation of CASP8 over time (Figure 6E,F), which was detected by pulling down the unstimulated receptors using FLAG-tagged TNFSF10/TRAIL added to the cellular lysates. Similarly, interference of FYCO1 expression resulted in a striking increase of TNFRSF10B/DR5, while the TNFRSF10A/TRAIL-R1/DR4 (TNF receptor superfamily member 10a) levels were not significantly changing (Figure 6G).

### FYCO1 regulates TNF receptor complex turnover

**Figure 7.**
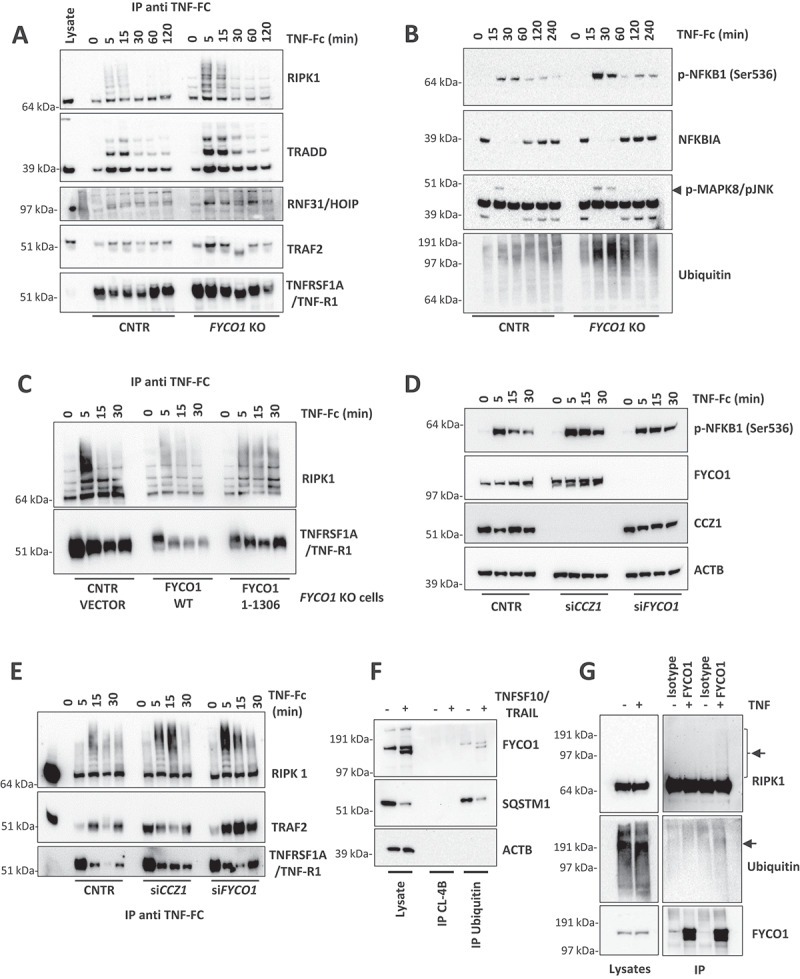
FYCO1 regulates TNF receptor complex turnover. (A) the TNFRSF1A/TNF-R complex was isolated from control and FYCO1 KO HeLa cells treated with TNF-Fc containing supernatant for different time points. Shown is the WB analysis for the proteins indicated. (B) WB analyses of the full cell lysates prepared as in (A). (C) WB analyses of the TNFRSF1A/TNF-R complexes obtained from FYCO1 KO HeLa cells transfected with empty vector, FYCO1 WT or its truncated (1–1306) mutant, treated with TNF-Fc containing supernatant for 5-15-30 min. (D) WB for p-NFKB1 levels in HeLa cells transfected with siRNAs against CCZ1 or FYCO1 (40 nM, 72 h). (E) Cells were treated as in (D) and then subjected to immunoprecipitation of the TNFRSF1A/TNF-R complex. Shown is an immunoblot for the proteins indicated. (F) Lysates of untreated and TNFSF10/TRAIL-treated cells (500 ng/ml, 4 h) were immunoprecipitated with ubiquitin-immobilized agarose and with control CL-4B Sepharose. Specific binding of FYCO1 and SQSTM1/p62 was visualized by WB. (G) WB analyses of anti-FYCO1 co-IPs in lysates of cells untreated or treated with TNF-Fc containing supernatant for 5 min.

These results prompted us to investigate whether the stabilization and more efficient formation of the TNFSF10/TRAIL receptor complex could be shared by TNF receptors since their rapid internalization and lysosomal degradation were also reported to occur [30,31]. Therefore, we stimulated control and *FYCO1* KO cells with TNFα-Fc for different time points and then performed immunoprecipitations of the TNF-receptor complex. In cells with *FYCO1* KO, we observed a striking increase of associated proteins, including TRADD (TNFRSF1A associated via death domain), TRAF2 (TNF receptor-associated factor 2), polyubiquitinated RIPK1 and the Linear ubiquitin chain assembly complex (LUBAC) protein RNF31/HOIP (ring finger protein 31) (Figure 7A). In control cells TNFRSF1A/TNF-R1 levels decreased rapidly upon TNF treatment, to be then reconstituted after 60 min because of new transcription. Differently, in *FYCO1* KO cells the levels remained stable over time, suggesting a defect in TNFRSF1A/TNF-R1 degradation (Figure 7A). The more efficient formation of the TNF receptor complex resulted in stronger activation of downstream inflammatory pathways, manifested by increased phosphorylation levels of MAPK8/JNK (mitogen-activated protein kinase 8) and NFKB1 (nuclear factor kappa B subunit 1) (Figure 7B), and by the stronger re-expression of the NFKB1 target NFKBIA/IKBα (NFKB inhibitor alpha) (Figure 7B). Noteworthy, in *FYCO1* KO cell lysates, we detected a striking increase of polyubiquitinated proteins upon short exposure to TNF (Figure 7B).

In line with our described model, reconstitution of KO cells with truncated FYCO1^1–^^1306^ was not able to rescue the accumulation of the TNFRSF1A/TNF-R1 complex as full-length FYCO1 did (Figure 7C). In agreement, both CCZ1 and FYCO1 KD by siRNAs were associated with prolonged NFKB1 phosphorylation (Figure 7D), because of stronger and stabilized TNFRSF1A/TNF-R1 complex formation (Figure 7E). To dissect the mechanisms of FYCO1 involvement in TNF signaling, we aimed to verify the presence of a direct interaction of the protein with any component of the complex, other than CASP8. Since FYCO1 deficiency was associated with an accumulation of polyubiquitinated proteins, we supposed and validated its capacity to bind to ubiquitin, as demonstrated by the specific immunoprecipitation of FYCO1 by ubiquitin-conjugated beads (Figure 7F). Given that RIPK1 is a well-established polyubiquitinated protein, we hypothesized a direct interaction between the two proteins. We confirmed this finding by reciprocal co-immunoprecipitation of the ectopically expressed tagged proteins in 293T cells (**Figure S7A-B**), as well as by detection of endogenous ubiquitinated RIPK1 in anti-FYCO1 co-IP of HeLa cells treated with TNF (Figure 7G).

### Proposed working model

**Figure 8.**
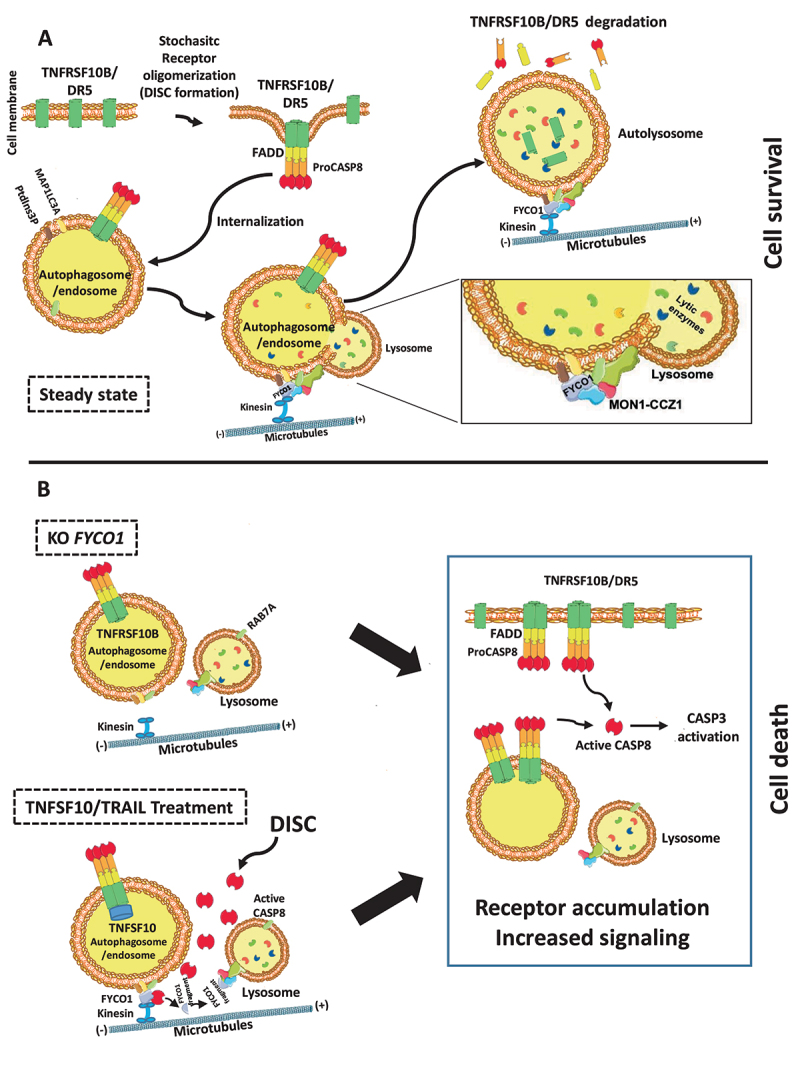
Proposed working model. (A) FYCO1 mediates efficient lysosome-mediated removal of stochastic CASP8 auto-activating complexes, and this is necessary to ensure cell survival. The fusion of endocytosed complexes with lysosomes is dependent on the interaction of the C-terminal GOLD domain of FYCO1 with the RAB7A GEF complex containing CCZ1-MON1A, further responsible for interaction with the HOPS complex. (B) In absence of FYCO1, the autolysosomal degradation of receptors is impaired and the accumulating receptors sensitize the cells for ligand-induced and constitutive apoptosis. Furthermore, if a certain stimulus threshold is reached (e.g., by addition of TNFSF10/TRAIL), CASP8-mediated cleavage of FYCO1 C-terminal domain prevents this lifeguard mechanism, thus allowing amplification of the apoptotic cascade and execution of cell death.

In summary, the neutralization of FYCO1 resulted in a more efficient and stable formation of both TNFSF10/TRAIL and TNF receptor complexes, with prolonged and enforced signaling and downstream apoptotic or pro-inflammatory effects. This was the result of impaired lysosomal degradation of endocytosed complexes. The late endosome-lysosome fusion is dependent on the interaction of the C-terminal GOLD domain of FYCO1 with the RAB7A GEF complex containing CCZ1-MON1A, further responsible for interaction with the HOPS (homotypic fusion and protein sorting) complex [25]. Our data suggested that the efficient removal of stochastic CASP8 auto-activating complexes was necessary to ensure cell survival (Figure 8A). Over a certain stimulus threshold, CASP8-mediated cleavage of the FYCO1 C-terminal domain prevented this lifeguard mechanism and instead resulted in the accumulation of the receptors and consequently in the amplification of the apoptotic cascade and the execution of cell death (Figure 8B). This threshold was reached by stochastic receptor oligomerization in the absence of FYCO1 (KO) or by forced DISC formation upon treatment with TNFSF10/TRAIL.

## Discussion

FYCO1 functions in the autophagic pathway by regulating the bidirectional transport of autophagosomes and endosomes through the microtubules and is required for efficient maturation of the vesicles [9,23]. Interestingly, mutations of FYCO1 are associated with autosomal-recessive congenital cataracts [32], a major cause of vision loss in children, and fyco1 homozygous knockout mice recapitulate the cataract phenotype consistent with a critical role of this protein in autophagic flux, organelle removal, and lens morphogenesis [33].

Here we describe FYCO1 as a novel interactor of activated CASP8 and show that loss of FYCO1 function resulted in apoptosis induction and sensitized surviving cells to death induced by CASP8 activating stimuli. The sensitization correlated with an accumulation of TNFRSF10B/DR5 and a more effective TNFSF10/TRAIL DISC formation. Loss of FYCO1 was associated with a reduced colocalization of TNFRSF10B/DR5 with lysosomes, suggesting a block of the lysosomal degradation of the DISC complex (see the model in Figure 8). Our data add another piece of evidence of how caspase activation may influence the function of proteins known to be implicated in vesicle transport. Caspases can cleave autophagic proteins, inactivating them or turning them to co-activators of apoptosis [34–38]. As an example, BECN1 (beclin 1), a key regulator of autophagy, is cleaved by caspases within chemotherapy-induced and mitochondria-mediated apoptosis. Perturbation of BECN1 cleavage contributes to chemotherapy resistance *in vitro* and *in vivo* [39,40]. Interestingly, the C-terminal fragment of BECN1, generated by the caspase-mediated cut, is localized in the mitochondria, and sensitizes cells to apoptosis, probably through the release of pro-apoptotic factors [41].

Noteworthy, we demonstrated that FYCO1 is cleaved at Asp1306 by activated CASP8, but to a significantly lesser extent by effector CASP3. These findings are similar to results that were shown before for other bona fide CASP8 substrates such as BID (BH3 interacting domain death agonist) and RIPK1, underlining the well-established substrate overlap of the two enzymes [42]. This was an interesting finding, as few specific substrates of initiator CASP8 are known, pointing toward a functional impact of this cleavage. We indeed observed that only the reconstitution of *FYCO1* KO cells with a non-cleavable version of the protein reduced DISC formation and inhibited apoptosis. The C-terminal fragment generated by CASP8 cleavage roughly corresponds to the GOLD domain of FYCO1. Recently, Liu et al. reported that the GOLD domain interacts with the CCZ1-MON1A complex [43] that regulates endosome/autophagosome maturation and fusion with lysosomes [24–28]. We showed that a truncated FYCO1 mutant lacking the C-terminal cleavage fragment was not interacting with CCZ1 and that the knockdown of CCZ1 was phenocopying the effects of FYCO1 silencing. Interestingly, ectopic expression of the C-terminal fragment of FYCO1 (aa 1307-end) interfered with the interaction of the two proteins. FYCO1 C terminus (aa1307-end) did not localize to mitochondria, but acted as a dominant negative mutant, resulting in an increase of the autophagic marker LC3B-II, accumulation of TNFRSF10B/DR5, and auto-activation of CASP8. These results indicated that the release of the C-terminal fragment of FYCO1 upon its cleavage might not only inactivate its anti-apoptotic function but also rather enhance the amplification of the apoptotic cascade.

Our results provide a molecular mechanism that helps to better understand previous findings describing a general protective role of the endocytotic machinery against TNFSF10/TRAIL-induced apoptosis [44–49]. TNFSF10/TRAIL and its receptors are rapidly endocytosed in a time- and concentration-dependent manner and this internalization is mediated by clathrin-dependent and independent mechanisms; blockade of receptor internalization was reported to amplify apoptotic signaling of TNFSF10/TRAIL [50–52]. Expression of death receptors TNFRSF10A/DR4 and TNFRSF10B/DR5 on the surface of cell membranes is a critical determinant of cancer cell sensitivity to recombinant TNFSF10/TRAIL and to its receptor agonistic antibodies, and their mislocalization in intracellular compartments has been shown to be an important resistance mechanism [53].

Finally, we extended our results to the assembly and activation of the TNF receptor complex, demonstrating that in the absence of FYCO1 the stimulation with TNF leads to a facilitated activation of downstream anti-apoptotic and inflammatory pathways. Given the pleiotropic role of TNF in cellular biology and in organisms’ physiology and disease, this finding paves the way for multiple fascinating avenues of research. Interestingly, FYCO1 is one of the six genes of the 3p21.31 cluster that two independent genome-wide association studies/GWAS [54,55] identified as a genetic susceptibility locus in patients with respiratory failure from severe COVID-19 [56].

## Materials and methods

### Cell culture

HEK293T, HeLa and HCT116 cells were purchased from ATCC (CRL-11268, CCL-2 and CCL-247). Cells were grown respectively in Iscove Modified Dulbecco Medium IMDM (Euroclone, ECB2072L), RPMI 1640 (Euroclone, ECM2001L) and McCoy’s (GIBCO, 36600–021) plus 10% fetal bovine serum, FBS (Euroclone, ECS0180L) and antibiotics (Euroclone, ECB3001D) at 37°C and 5% CO_2_.

### FASLG, TNFSF10/TRAIL and TNF production

TNF-Fc, HF-TNFSF10/TRAIL and FASLG-Fc were expressed and purified as described before [15,57,58]. In short, the cDNAs encoding the extracellular portion of human TNF (AA78–233) or human FASLG/CD95L (AA117–281) were fused at the N terminus to the constant region (Fc) of human IgG1 (CH2-CH3, AA102–329), preceded by the TNFRSF10B signal peptide (AA1–55). The expression vector pcDNA3.1 (Invitrogen, V79020) was transfected in HEK293T cells and the supernatants containing the Fc-tagged proteins were used for the experiments shown. HF-TNFSF10/TRAIL was produced by cloning the extracellular portion of human TNFSF10/TRAIL (AA95–281) in the pQE32 vector (Qiagen, N32915) preceded by a FLAG and 6×His tag. The protein was expressed in M15 bacteria (XL Biotech, CHC00032) and purified using Ni-NTA (Cytiva, 29048631).

### bTIP (bridged two-step IP) for the identification of CASP8-binding proteins

For mass spectrometry analysis, we performed three independent bTIP preparations according to the protocol described in Sciuto et al [16]. Each experiment consisted of three samples, starting from 5 × 10^7^ HeLa cells/sample. The cells were either left untreated or treated with HF- TNFSF10/TRAIL (the recombinant protein was produced as explained above) at 1 µg/ml for 3 h; then washed with cold PBS (Euroclone, ECB4004L) and lysed in IP-lysis buffer for 30 min at 4°C on a rotator. IP lysis buffer was prepared with 30 mM Tris-HCl, pH 7.4, 120 mM NaCl, 10% glycerol, 2 mM EDTA, 2 mM KCl, 1% NP40 (Sigma Aldrich, I3021) and supplemented with protease inhibitors (Complete EDTA-free protease inhibitor; Roche, 4693132001). The lysates were cleared by centrifugation at 16,000 g (4°C, 30 min). Untreated cells were either incubated with 2 µg anti-CASP8 C20 antibody (Santa Cruz Biotechnology, sc-6136) or with 2 µg goat IgG (ProSci, 3705); lysates from treated cells were incubated with anti-CASP8 antibody; 4 µg of biotin-conjugated secondary anti-goat antibody and anti-biotin beads (Sigma Aldrich, A1559) were added to each sample. The first step of precipitation was allowed overnight at 4°C on a rotator. After 5 washes of the beads in IP-lysis buffer, the proteins bound to the anti-biotin beads were eluted by two consecutive elution steps in biotin buffer reaching a final biotin concentration of 3.3 mM in IP-lysis buffer. After each elution, the samples were centrifuged (900 g, 4°C, 3 min) and the supernatant was carefully transferred to a new tube. The second co-immunoprecipitation was performed with Protein G beads (GE Healthcare, 17-0618-01) at 4°C using a rotator for 4 h. Afterwards the beads were washed 5 times and stored at −80°C until analysis.

### Sample preparation for MS analysis

Proteins from Co-IP beads were resolved in LDS sample buffer (Thermo Fisher Scientific, NP0007) and short (2 cm) gel electrophoresis runs were performed for the sample cleanup. Proteins were digested in-gel as previously described [15]. Briefly, gel bands were sliced, protein reduction and alkylation was performed with 10 mM DTT at 27°C and 30 mM IAA for 1 h at RT and digested with trypsin:protein 1:50 (Promega, VA9000) over night at 37°C. Prior to MS analysis, acetonitrile (MeCN):H_2_O, 50:50 (v:v), gel extracted peptides were combined, desiccated and redissolved in water, containing 2.5% 1,1,1,3,3,3-hexafluoro-2-propanol (HFIP) and 0.1% trifluoroacetic acid (TFA).

### nanoLC MS/MS analysis

Peptide samples were separated and analyzed by nanoflow LC-MS/MS. We used a Dionex 3000 nanoUHPLC (Thermo Fisher Scientific) in line with an Orbitrap Exploris mass spectrometer (Thermo Fisher Scientific). Peptide loading and washing was performed with 0.1% TFA in water for 3 min at a flow rate of 30 μl/min using a trapping cartridge Acclaim PepMap300 C18, 3 μm, 300Å (Thermo Fisher Scientific, 060263). Peptides were separated on a nanoEase, 1.7 μm, 300Å, 75 μm x 200 mm analytical column (Waters, 186008800) at a flow rate of 300 nl/min. A three step 90 min gradient consisting of the following steps was applied for nanoLC separation: 2–4% solvent B (99.9% MeCN, 0.1% formic acid (FA)) in 4 min, 4–30% in 76 min and 30–80% in 3 min followed by a washing and an equilibration step with 0.1% FA in water. The spray voltage was 2.2 kV for nanoESI ionization and the ion transfer tube temperature was set to 275°C. The MS instrument was operated in the data-dependent (DDA) mode. Full scan MS spectra (m/z 380–1,400) were acquired with a maximum injection time of 45 ms at 60,000 resolution and an automatic gain control (AGC) target value of 300%. The normalized MS collision energy was set to 26. MS/MS scans cycles were triggered for 1.5 sec. High-resolution MSMS spectra were acquired in the orbitrap with a maximum injection time of 22 ms at 15,000 resolution. An isolation window of 1.4 m/z and an AGC target value of 100% was set for MSMS analyses. Dynamic exclusion was set to 30 s. Undetermined charge states and single charged signals were excluded from MS/MS fragmentation.

### Peptide and protein identification and quantification

MS raw data processing and statistical analysis was performed using MaxQuant v2.0.1.0 software package including Perseus v1.6.15.0 [59,60]. For protein identification the UniProt database UP000000589 (Homo sapiens; 01, 2020; 20367 sequences) was applied. Carbamidomethylation of cysteines was set as fixed modification. N-terminal acetylation, glutamine, and asparagine deamidation and methionine oxidation were set as variable modifications. Identification FDR cutoffs were 0.01 on the peptide level and on the protein level. The “match-between-runs” function and LFQ option were enabled for label free quantification. Proteins were identified with a FDR ≤ 1%. A two-sample *t*-test (*p* value: ≤ 0.01) was performed to define significantly regulated proteins between TNFSF10/TRAIL stimulated and unstimulated WT samples.

### Co-IP and bTIP for protein interaction analysis

To perform the co-immunoprecipitation reactions, we used IP-lysis buffer (30 mM Tris-HCl, pH 7.4, 120 mM NaCl, 10% glycerol, 2 mM EDTA, 2 mM KCl, 1% NP40 with Protease inhibitors). The resins used were: protein G beads (17-0618-01) obtained from GE Healthcare; anti-biotin beads (A1559), anti-FLAG beads (A2220), anti-V5 beads (A7345) obtained from Sigma Aldrich; anti-rabbit beads (00-8800-25) from Rockland Bioscience; Ubiquitin, agarose immobilized (BML-UW8630–0500) obtained from Enzo. The antibodies used for co-immunoprecipitation were: anti-CASP8 (sc-6136) and anti-CCZ1 (sc-514290) obtained from Santa Cruz Biotechnology; anti-CASP8 (ALX-804-242-C100) from Enzo; anti-FYCO1 (PA5–45805 and HPA035526) obtained from Thermo Fisher Scientific and from Sigma Aldrich. Immunoprecipitation negative controls were obtained with rabbit IgG (3703) and goat IgG (3705) from ProSci; rabbit IgG (0111–01) from Southern Biotech; mouse IgG1, kappa monoclonal [MOPC-21] - isotype control (ab18443) from Abcam. Recombinant HF- TNFSF10/TRAIL was added to lysates for TNFRSF10/TRAIL-R complex immunoprecipitation of unstimulated cells with anti-FLAG beads. IP of TNFRSF1A/TNF-R complex was obtained with protein G beads capturing TNF-Fc used to stimulate cells or pre-coupled to beads for unstimulated cells.

### Western blot analysis

For protein lysates preparation, cells pellets were resuspended in RIPA buffer (150 mM NaCl, 20 mM Tris, pH 7.2, 0.05% SDS, 1.0% Triton X-100 [SigmaAldrich, X100], 1% deoxycholate [Sigma Aldrich, D6750], 5 mM EDTA [Merck 03609]) completed with protease and phosphatase inhibitor cocktails (Sigma Aldrich; P2714, P5726, P0044). The lysates were cleared by centrifugation (16000 g, 4°C, 20 min). Lysate fractionation was obtained by Qproteome Cell Compartment (Qiagen, 37502). Where indicated, the cells were pre-treated for 4 h with 500 nM rapamycin (Selleckchem, S1039). The lysates and bead bound proteins were treated with NUPAGE LDS sample buffer (Thermo FisherScientific, NP0008) under reducing conditions (50 mM TCEP; Thermo Fisher Scientific, 77720), separated by Bolt 4–12% Bis-Tris plus gradient gel (Thermo FisherScientific, NW04125) using MOPS or MES buffer and blotted to a nitrocellulose membrane (GE Healthcare, 10600018). The membrane was blocked for 1 h with TBST (20 mM Tris, 150 mM NaCl, 0.1% Tween 20 [Sigma Aldrich P1379]) containing 5% blotting grade nonfat powdered milk. The antibodies used for western blot were the following: antibodies for CASP8 (clone C-15) [61] and CFLAR/cFLIP (clone NF6) [61] were hybridoma supernatants obtained from Krammer’s lab (German Cancer Center (DKFZ) Heidelberg, Germany). Antibodies for CASP3 (9665), CASP7 (9494), cleaved CASP7 (9491), cleaved CASP8 (9496), cleaved PARP1 (9541), TNFRSF10A/DR4 (42533), TNFRSF10B/DR5 (8074), p-NFKB1 Ser536 (3033), NFKBIA (4814), TRAF2 (4724), LC3B (3868), ubiquitin (3936) and HSP90 (4877) were obtained from Cell Signaling Technology. Antibodies for PARP1 (sc-53643), TNFRSF1A/TNF-R1 (sc-8436), p-MAPK8/JNK (sc-6254), TIMM23 (sc-514463), CCZ1 (sc-514290), RAB7 (sc-376362), VPS41 (sc-377118) were obtained from Santa Cruz Biotechnology. Antibodies for FADD (610400), RIPK1 (610459) and TRADD (610572) were obtained from BD Biosciences. Antibodies for FYCO1 (HPA035526), TUBB/β-tubulin (T4026) and ACTB/β-actin (A5441) were obtained from Sigma Aldrich. Antibodies for V5 (MA5–15253) and GAPDH (MA515738) were obtained from Thermo Fisher Scientific. Rabbit polyclonal or mouse isotype-specific HRP-conjugated secondary antibodies were obtained from Southern Biotech.

### In vitro cleavage assay

To perform the *in vitro* cleavage assay, 293T were transfected with pcDNA 3.1 (Thermo Fisher Scientific, V79020) carrying wild-type or mutated V5-tagged FYCO1, by standard CaPO_4_ transfection methods. Anti-V5 immunoprecipitation was then performed from the lysates, resuspended in caspase reaction buffer (50 mM HEPES, 50 mM NaCl, 0.1% CHAPS [SigmaAldrich, 850500P], 10 mM EDTA, 5% glycerol and 10 mM DTT at pH 7.2) and aliquoted. Then, cleavage assays were performed by incubation at 37°C with indicated units of recombinant human CASP8 or CASP3 (Enzo; ALX-201-062 and ALX-201-059) for indicated timepoints. Part of the cleavage reaction was loaded onto SDS-PAGE gel for Coomassie Brilliant Blue staining with SimplyBlue™ SafeStain (Thermo Fisher Scientific, LC6060), another part subjected to WB with anti-V5 antibody. CASP8 and CASP3 activity was verified by Caspase-Glo 8 Assay (Promega, G8200) and Caspase-Glo 3/7 Assay (Promega, G8090).

### Generation of knockdown cells using shRNA

To produce the FYCO1 shRNA constructs, HEK293T were co-transfected with the packaging and pseudotyping DNAs (psPAX2, pMD2.G, obtained from the ADDGENE consortium [Addgene, 12260 and Addgene, 12259, respectively, deposited by the lab of Didier Trono]) and pLKO.1 lentiviral shRNA vectors encoding for control and *FYCO1*-targeting shRNAs. The FYCO1 targeting sequences TRCN0000038914, TRCN0000038915, TRCN0000038917, TRCN0000038918 in a pLKO.1-puro vector (respectively named pLKO #14, #15, #17, #18) were obtained from Sigma Aldrich. For transfection we used standard CaPO_4_ transfection methods according to the manufacturer’s recommendation (CalPhos; Clontech, NC9567834). For the generation of the inducible shRNAs, the TRCN0000038914 and TRCN0000038917 sequences containing the U6 promoter were subcloned into a lentiviral vector containing 5 repeats of the Tet-response elements in front of the *RNU6* promoter. The lentiviral vector was constructed by replacing the CMV-promoter of pLenti6/V5 D-TOPO (Invitrogen, K495510) by the Tet-response elements. The transgenic cells were generated by co-transduction with a lentiviral vector expressing the rtKRAB repressor. shRNA expression was induced with 250 nM doxycycline (Sigma Aldrich, D3447). Transient KD of FYCO1, CCZ1, RAB7A and VPS41 by siRNA was obtained with the following Silencer Select siRNAs from Ambion: s35794, s35795, s35796 (respectively named #94-#95-#96 for FYCO1), s230590, s230591 (respectively named #90-#91 for CCZ1), s15443, s15444 (respectively named #43-#44 for RAB7A), s25768, s25769 (respectively named #68-#69 for VPS41). Scramble negative control was AllStars Negative Control siRNA (QIAGEN, SI03650318). HeLa cells were transfected with HiPerFect (QIAGEN, 301704) reagent following manufacturer’s instructions. Briefly, cells were transfected with 40 nM oligos and 6 µl HiPerFect/ml. T47D cells were transfected with Dharmafect 1 (Dharmacon, *T*-2001) with 40 nM oligos and 2 µl Dharmafect/ml.

### Generation of *FYCO1* KO cells using CRISPR-Cas9

Four different oligonucleotide pairs for guide RNAs (Guide #2: GAACAGCCCCCTAAACAACG, Guide #4: GGGGCTGTTCAGGTCAAAGT, Guide #6: ATGCTGGAGCTGCGGCTAGG, Guide #11: GCTTTGACTTGGATGCTGCC) were designed and annealed as reported before [19] and cloned into the p×330vector. The vector pX330-U6-Chimeric_BB-CBh-hSpCas9 was a gift from Feng Zhang (Addgene, 42230; http://n2t.net/addgene:42230; RRID: Addgene_42230). Their capacity to reduce FYCO1 levels was first tested by transfection of 293T. Two plasmids (pX330 sgRNA Fyco1 #2 and p×330sgRNA Fyco1 #11) were then selected and transfected in HeLa and HCT116 cells in combination with a GFP-reporter in limited amounts. Transfection was performed with Fugene HD reagent (Promega, PRE 2311) according to manufacturer’s instructions. Transfected cells were sorted by Fluorescence Activated Cell Sorter (FACS) and single-cell cloned. Western blot was then used to verify *FYCO1* knockout.

### Analyses of CRISPR-mediated gene editing

To analyze the gene editing we amplified the genomic regions surrounding the sgRNA binding sites by PCR using the following oligonucleotides: G#11_For_1: GGTTTGTGTGCCTGTGTCTT; G#11_Rev_1: ATCTCCACTGACTCATGCCC; G#2_For_2: TCCCTCCCAGACTCAAGAGA and G#2_Rev_2: CATGTCTGCAGCTCCTTCAC. The PCR strategy is shown in supplementary figure S3E. In short, the sequences surrounding the sgRNA binding sites were amplified using a proofreading polymerase (Phusion; New England Biolabs, M0531S), gel purified, cloned by Blunt TOPO cloning using the pCR4 Blunt TOPO cloning kit (Invitrogen, 450031) using the manufacturers’ recommendations and 9–10 clones were sequenced by Sanger sequencing.

### Cytofluorimetric analysis of TNFSF10/TRAIL Receptors

Cells were stained 72 h post-transfection with 10 µg/ml monoclonal anti-TNFRSF10A/TRAIL-R1, TNFRSF10B/TRAIL-R2, TNFRSF10C/TRAIL-R3, TNFRSF10D/TRAIL-R4 (Adipogen; HS-101, HS-201, HS-301, HS-401, respectively) for 30 min at 4°C. After washing, cells were incubated for an additional 30 min at 4°C with 5 µg/ml Phycoerythrin (PE)-conjugated goat anti-mouse secondary antibody (Thermo Fisher Scientific, P-852), and after further washing analyzed with a CytoFLEX flow cytometer (Beckman Coulter).

### Cell viability assay

Cell viability was analyzed using Cell Titer-Glo® Luminescent Cell Viability Assay (Promega, 47571) and luminescence was analyzed with Perkin Elmer Victor X3.

### Immunofluorescence

For immunofluorescence analysis, cells attached on 15-mm polylysine-coated coverslips placed in a 24-well plate were first pre-incubated with bafilomycin A_1_ (Sigma Aldrich, B1793) 100 nM for 1 h to prevent lysosomal acidification, then treated with TNFSF10/TRAIL at 1 µg/ml for 2 h. Cells were fixed with 4% paraformaldehyde for 15 min and permeabilized with PBS containing 0.1% Triton X-100 for 3 min at room temperature. Slides were incubated overnight at 4°C with anti-LAMP1 (Santa Cruz Biotechnology, sc-20011) and anti-TNFRSF10B/DR5 (Cell Signaling Technology, 8074) diluted 1:50 each in PBS containing 3% bovine serum albumin (Sigma Aldrich, A4503), 0.05% Triton X-100. After extensive washing, coverslips were incubated for 45 min at room temperature with donkey anti-mouse Alexa Fluor 488-conjugated and donkey anti-rabbit Alexa Fluor 555-conjugated secondary antibody (Thermo Fisher Scientific, A-21202 and A-31572), while DAPI (4′, 6′-diamidino-2-phenylindole) was used for nuclear staining. Coverslips were mounted on microscope slides and sealed. Images were taken on a confocal microscope Zeiss LSM 900 (Carl Zeiss GmbH, Jena, Germany) in Airyscan mode. Excitation light was obtained by diode lasers: 405, 488, 561. Optical thickness was 0.20 µm with 63× objective. Images have been treated and analyzed by the Zen Blue (3.2) software (Carl Zeiss GmbH, Jena Germany) and ImageJ (1.53) software. For each slide, 10 fields were acquired. Generated signal dotplots were adjusted by applying similar cutoff values, to obtain values for TNFRSF10B/DR5 (555) and LAMP1 (488) pixel colocalization. The percentage of colocalized TNFRSF10B/DR5 was calculated over the total TNFRSF10B/DR5 signal and was compared among samples by Student t-test.

### Site-directed mutagenesis

The presence of consensus sequences for CASP8 cleavage on FYCO1 was verified through the PROSPER (Protease specificity prediction server; https://prosper.erc.monash.edu.au/) program on which the amino acid (aa) sequence of FYCO1 was launched starting from aa1086 (the C-terminal part of the protein). Two potential sites were identified, corresponding to the aspartates in aa position 1238 and 1306. A fragment of about 900 base pairs containing the two potential sites and containing a 5’-EcoRI (in the FYCO1 cDNA) and an XbaI site (in the pcDNA3.1 cDNA) was transferred into pCRII vector, yielding a pCR II-Fyco1 fragment that served as an intermediate vector. Polymerase Chain Reaction (PCR) was then performed with primers bearing the mutation of aspartate with alanine for aa 1238 and 1306 using Phusion® High-Fidelity PCR Master Mix (NEB, M0531S). After digestion with DpnI, the mutated fragments were transferred back to the original pcDNA3.1 vector (Thermo scientific) containing the remaining part of FYCO1. For the generation of the FYCO1^F1280A,I1283A^ mutant a similar strategy was applied. We used two oligonucleotides with the desired base exchanges in the LIR domain of the protein (LIRmut_rev: atcagcCACAGCGTCGTCCGGTG and LIRmut_for: atcgcCACAGATGAGGAATTGTGCCAGATAC) and performed a PCR amplifying the whole plasmid using Phusion® High-Fidelity PCR Master Mix (NEB, M0531S). The mutation was then validated by Sanger sequencing and the mutated *FYCO1* fragment was cloned again in the pcDNA3.1 vector containing the remaining *FYCO1* cDNA.

### Cloning of the C-terminal FYCO1 fragment

For the cloning of the C-terminal FYCO1 fragment, the sequence encoding for aa1306 to aa1479 was amplified from the pcDNA3.1 vector by PCR including a PmeI and XhoI restriction site. The cDNA was transferred in a pLenti6 containing 5 repeats of the Tet-response element (see above). The cells were then co-infected with an expression construct for the rtTA3 (Tet-activator) and the expression of the transgene was induced by addition of doxycycline at 250 ng/ml.

### Statistical analysis

Data shown represent 3–5 independent biological experiments. Statistical analyses were conducted by using two-way ANOVA and by unpaired Student’s t test *p < 0.05; **p < 0.01; ***p < 0.001 with Prism (GraphPad) software.

### Database entry for the mass spectrometry results

The mass spectrometry proteomics data have been deposited to the ProteomeXchange Consortium via the PRIDE partner repository with the dataset identifier PXD033760.

## Supplementary Material

Supplemental Material
