# Supplementary material for: The autophagic protein FYCO1 controls TNFRSF10/TRAIL receptor induced apoptosis and is inactivated by CASP8 (caspase 8)
Source: Autophagy. 2023 Jul 7;19(10):2733–51. doi: 10.1080/15548627.2023.2229656 (PMC10472876; doi:10.1080/15548627.2023.2229656)
Supplement: Supplemental Material [file KAUP_A_2229656_SM7254.zip › Coppola et al_suppl. figure 1-7_R5_no track.docx]

**Figure S1**. Validation of FYCO1 as an interactor of activated CASP8. **(A)** HeLa cells were treated with HF-TNF (4 h), Fc-FASLG/CD95L (4 h), or TNFSF10/TRAIL (2 h) in the presence of cycloheximide (CHX, 1 µg/ml) and anti-CASP8 co-IP was performed on lysates. Shown is the WB for the proteins indicated. **(B)** Representative reverse co-IP with anti-FYCO1 antibody. Lysates of HeLa cells treated with TNFSF10/TRAIL for 2 h were co-immunoprecipitated with polyclonal anti FYCO1 antibody or rabbit IgG. In the WB the p18 fragment of activated CASP8 was detected (indicated by the arrow). **(C)** Luminescence-based determination of recombinant CASP8 and CASP3 activity at indicated time points.

**Figure S2.** The absence of FYCO1 facilitates basal and CASP8-induced apoptosis. (**A**). Representative microphotographs showing apoptotic membrane blebbing upon FYCO1 KD by shRNA, and its reversal by Z-VAD-FMK treatment. (**B**) Cell viability assay (Cell titer GLO, Promega) of HeLa cells 72 h after transfection with scramble oligo or siRNA FYCO1 #95 and #96, in the presence or not of Z-VAD-FMK (20 µM) or Z-IETD-FMK (10 µM). Mean ± standard error of 3 independent experiments performed in triplicate, statistical analysis by Student t-test. (**C**) WB for the proteins indicated in HeLa cells knocked down for FYCO1 expression with 2 different siRNAs (40 nM, 72 h) and then treated with different stimuli (TNFSF10/TRAIL 500 ng/ml for 2 h; FcFASLG/CD95L SN 1:100, Poly IC 5 µg/ml and recombinant TNF 1 µg/ml for 4h). (**D+E**). The absence of FYCO1 sensitizes T47D cells for TNFSF10/TRAIL-induced apoptosis. (**D**) WB for the proteins indicated in T47D cells knocked down for FYCO1 expression with 2 different siRNAs (#95 and #96; 40 nM, 72 h) and two different shRNA constructs (#14 and #18) and then treated with TNFS10/TRAIL (2 µg/ml for 4 h). (**E**) Cell viability assay (Cell titer GLO, Promega) of T47D cells transduced with control vector (Cntr si) or two different lentiviral constructs encoding FYCO1 shRNA constructs (#14 and #18). Cells were treated for 24 hours with 1 µg/ml TNFSF10/TRAIL and the results are displayed as mean ± standard error of 3 independent experiments performed in triplicate, statistical analysis was performed using Student t-test. (**F**) WB for the proteins indicated shows enhanced caspase activation in 2 different clones of CRISPR/Cas9-mediated FYCO1-knocked out HeLa cells, treated for different timeframes with TNFSF10/TRAIL (500 ng/ml).

**Figure S3.** Validation of results in another cellular model. (**A**) HCT116 cells were transduced with two inducible FYCO1 shRNA constructs (ind. sh#14 and sh#17). KD was induced by doxycycline treatment (250 ng/ml, 48-72- 96 h) and WB was performed to detect the proteins indicated. (**B**) WB validation of the CRISPR-Cas9-mediated *FYCO1* KO in HCT116 clones. (**C**) Cell viability assay of control and *FYCO1* KO HCT116 cells upon TNFSF10/TRAIL treatment for 24 h. Reported is the mean ± standard error of viability obtained in 3-5 independent experiments. Statistical analysis was performed by two-way ANOVA with Dunnett test vs WT cells. (**D**) 2 different HCT116 *FYCO1*-KO clones were treated with TNFSF10/TRAIL (500 ng/ml) for the indicated time frames and WB for the indicated proteins was performed. **(E)** The CRISPR-mediated gene editing was controlled by PCR amplification of the genomic loci encoding for FYCO1, subsequent TOPO cloning of the PCR products, and Sanger sequencing of 9-10 clones obtained. The upper part of the figure shows the CRISPR sgRNA target sites, the PCR primers used (see materials and methods), and the expected length of the PCR product. The table shows the frequency of mutations observed upon gene editing and error-prone repair. Please consider that HCT116 cells are diploid for the *FYCO1* locus, while HeLa cells are pseudo-tetraploid.

**Figure S4.** Ectopic expression of an LIR-deficient FYCO1 mutant does not significantly change TNFSF10/TRAIL sensitivity in *FYCO1* KO cells. (**A**) Schematic representation and sequence confirmation of the FYCO1 mutant with defective LIR (FYCO1^(F1280A/I1283A)^) by Sanger sequencing. (**B**) HeLa *FYCO1* KO cells were either transfected with a pCDNA3.1 vector (vec) or a pcDNA3.1-based expression construct for FYCO1^F1280A,I1283A^ (dLIR). After 30 h cells were treated with TNFSF10/TRAIL for 120 min and immunoblot for the proteins described in the figure was performed.

**Figure S5.** Expression of the receptors for TNFSF10/TRAIL upon knockdown for FYCO1, RAB7A, and VSP41. (**A**) Flow cytometric analyses for the receptors indicated in HeLa cells knocked down for FYCO1 expression with 2 different siRNAs (#95 and #96; 40 nM, 72 h). Shown is a representative experiment. The values in the histograms represent the MFIs. (**B**) WB for TNFRSF10B/DR5 expression in T47D cells transfected with 2 different siRNAs for FYCO1 (40 nM, 72h). (**C**) WB for the proteins indicated in HeLa cells knocked down for RAB7A and VPS41 expression with 2 different siRNAs, each (40 nM, 72 h). (**D**) Knockdown of RAB7A and VPS41 sensitizes HeLa cells for TNFSF10/TRAIL-induced death. Cell viability assay (Cell titer GLO, Promega) of HeLa cells transfected with the indicated siRNAs (40 nM, 72 h). Cells were treated for 24 hours with 100 ng/ml TNFSF10/TRAIL and the results are displayed as mean ± standard error of 5 independent experiments performed in triplicate, statistical analysis was performed using the Student t-test. (**E+F**). The absence of RAB7A and VPS41 increases TNFRSF10B/DR5 surface expression. Shown is a representative experiment of flowcytometric analyses for TNFRSF10B/DR5 indicated in HeLa cells knocked down for RAB7A (E) and VPS41 (F) expression with 2 different siRNAs (40 nM, 72 h). The values represent the MFIs.

**Figure S6.** FYCO1 loss of function leads to the accumulation of LC3B and its C-terminal fragment acts as a dominant negative. (**A**) HeLa cells were transduced with a control vector or an inducible expression construct for the C-terminal FYCO1 fragment (FYCO1 1307-end). FYCO1 1307-end (500 nM, 5 h) and/or bafilomycin A_1_ (200 nM, 5 h), lysed and WB analyses for LC3B was performed. (**B**) Control and *FYCO1* KO HeLa cells were treated with autophagy inducer rapamycin (500 nM, 5 h) and/or bafilomycin A_1_ (200 nM, 5 h) in the presence of 1% FBS. Then the cells were lysed and WB analyses for the indicated proteins were performed. (**C**) WB analyses showing cytoplasmic localization of the FYCO1 C-terminal fragment in fractionated lysates of *FYCO1* KO HeLa cells transfected with pcDNA Fyco1-V5, treated with TNFSF10/TRAIL to obtain FYCO1 cleavage.

**Figure S7.** Reciprocal co-IP of FYCO1 and RIPK1. (**A**) 293T were transfected with pcDNA expressing MYC-tagged RIPK1, alone or in combination with V5 tagged FYCO1. Co-IP with anti-V5 beads and WB for the proteins indicated were performed. (**B**) 293T were transfected with pcDNA expressing V5 tagged FYCO1, alone or in combination with MYC-tagged RIPK1. Co-IP with anti-MYC beads and WB for the proteins indicated were performed.
