# Supplementary material for: The autophagic protein FYCO1 controls TNFRSF10/TRAIL receptor induced apoptosis and is inactivated by CASP8 (caspase 8)
Source: Autophagy. 2023 Jul 7;19(10):2733–51. doi: 10.1080/15548627.2023.2229656 (PMC10472876; doi:10.1080/15548627.2023.2229656)
Supplement: Supplemental Material [file KAUP_A_2229656_SM7254.zip › Table S2 R3.docx]

**Table S2.** Abundance of proteins enriched by CASP8-affinity isolation in the CRAPome database.

| **Mapped Gene Symbol** | **Num of Expt. (found/716)** | **Ave SC** | **Max SC** | **% of Expt.** |
| --- | --- | --- | --- | --- |
| *TNFSF10* | 0 | 0 |  | 0.0 |
| *TNFRSF10B* | 1 | 1 | 1 | 0.1 |
| ***FYCO1*** | **5** | **1** | **1** | **0.7** |
| *KIF21A* | 22 | 1.1 | 2 | 3.1 |
| *NACA* | 302 | 3.2 | 14 | 42.2 |
| *ATP2A2* | 302 | 4.2 | 41 | 42.2 |
| *CAPZB* | 366 | 7 | 72 | 51.1 |
| *HNRNPA3* | 447 | 6.6 | 49 | 62.4 |
| *SYNCRIP* | 459 | 7.5 | 97 | 64.1 |
| *HIST1H2BB* | 513 | 16.6 | 139 | 71.6 |
| *HNRNPA1* | 552 | 12.8 | 72 | 77.1 |

Shown are the proteins significantly enriched in the CASP8 pulldown (TNFSF10/TRAIL-stimulated vs non-stimulated cells) and their probability to be randomly identified (CRAPome database, 01.02.2023).
